# Gaseous Gangrene and Chillblains

**Published:** 1915-02

**Authors:** 


**Gaseous Gangrene and Chillblains.** G. Trifonoff, "Jour. de Med. et de Chir.," Montreal, December, 1914. His experience in the Balkan War included many cases of the former, with deep necrosis which he considered largely due to gas pressure. Treatment consisted in deep incision, abundant use of hydrogen peroxid, 12 volumes for preliminary washing, 8 volumes for dressings. Collargol ½ to 1 to 1000 was used in some cases. The cases mainly involved the lower extremities. Of 89 severe cases of chillblains, 85 resulted in gangrene from "congelation." The treatment included prolonged use of hot peroxid or permanganate solutions, tincture of iodine diluted 1 to 15 on the surrounding skin, and various astringent and antiseptic application, such as silver nitrate 2%, balsam of Peru 20% to 30%, red precipitate 4 per cent. to 5 per cent. incorporated with olive oil, chloroform, subnitrate of bismuth and vaseline.

